# *Clostridium perfringens* Delta-Toxin Damages the Mouse Small Intestine

**DOI:** 10.3390/toxins11040232

**Published:** 2019-04-22

**Authors:** Soshi Seike, Masaya Takehara, Keiko Kobayashi, Masahiro Nagahama

**Affiliations:** 1 Laboratory of Molecular Microbiological Science, Faculty of Pharmaceutical Sciences, Hiroshima International University, Kure, Hiroshima 737-0112, Japan; s-seike@ps.hirokoku-u.ac.jp; 2 Department of Microbiology, Faculty of Pharmaceutical Sciences, Tokushima Bunri University, Yamashiro-cho, Tokushima 770-8514, Japan; mtakehara@ph.bunri-u.ac.jp (M.T.); kobakei@ph.bunri-u.ac.jp (K.K.)

**Keywords:** *C. perfringens* delta-toxin, fluid accumulation, intestinal damage, E-cadherin

## Abstract

*Clostridium perfringens* strains B and C cause fatal intestinal diseases in animals. The secreted pore-forming toxin delta-toxin is one of the virulence factors of the strains, but the mechanism of intestinal pathogenesis is unclear. Here, we investigated the effects of delta-toxin on the mouse ileal loop. Delta-toxin caused fluid accumulation and intestinal permeability to fluorescein isothiocyanate (FITC)-dextran in the mouse ileal loop in a dose- and time-dependent manner. Treatment with delta-toxin induced significant histological damage and shortening of villi. Delta-toxin activates a disintegrin and metalloprotease (ADAM) 10, leading to the cleavage of E-cadherin, the epithelial adherens junction protein, in human intestinal epithelial Caco-2 cells. In this study, E-cadherin immunostaining in mouse intestinal epithelial cells was almost undetectable 1 h after toxin treatment. ADAM10 inhibitor (GI254023X) blocked the toxin-induced fluid accumulation and E-cadherin loss in the mouse ileal loop. Delta-toxin stimulated the shedding of intestinal epithelial cells. The shedding cells showed the accumulation of E-cadherin in intracellular vesicles and the increased expression of active caspase-3. Our findings demonstrate that delta-toxin causes intestinal epithelial cell damage through the loss of E-cadherin cleaved by ADAM10.

## 1. Introduction

Delta-toxin is a β-pore-forming-toxin (β-PFT) produced by *Clostridium perfringens* strains B and C [1]. While it is thought that delta-toxin may be implicated in necrotic enteritis in domestic animals and humans [1,2,3,4], the precise pathogenetic mechanism of action of the toxin is not clear. Delta-toxin hemolyzes the red blood cells of pigs, goats, and sheep [1,5,6]. Moreover, the toxin exhibits cytotoxic activity against multiple cell types, including macrophages, monocytes, and platelets from various animal species [1,7,8]. Delta-toxin has been assigned to the β-PFT family, which also includes alpha-toxin from *Staphylococcus aureus* and beta-toxin and NetB toxin from *C. perfringens* [9,10]. The structure of delta-toxin resembles alpha-toxin and NetB toxin [11]. According to structural analysis, delta-toxin forms a mushroom-shaped heptameric pore similar to that of alpha-toxin from *S. aureus* [11]. It is generally assumed that delta-toxin has the same mechanism of action as alpha-toxin.

It has been reported that ganglioside GM2 on the cell membrane plays a role in delta-toxin-induced cytotoxic effects [6]. Delta-toxin caused the death of GM2-expressing cells [6], but also generates an anion channel pore in planar lipid bilayers [9]. It has been indicated that the toxin also associate with other membrane constituents, although not with GM2 [9]. We reported that delta-toxin caused the rapid cell necrosis of sensitive cells, and that delta-toxin assembled into a toxic oligomer, which was associated with the cytotoxic activity, in cell membrane lipid rafts of susceptive cells [12]. Moreover, the toxin impaired permeabilization of mitochondrial membranes and the release of cytochrome *c* [12].

Investigations utilizing the isogenic beta-toxin null mutant of *C. perfringens* type C indicated that beta-toxin is necessary for type C strain-induced intestinal pathogenesis [3,13]. However, the possible participation of other toxins produced by type C strains is supported [1,4,10]. Delta-toxin is a virulence factor for type C strains [1,5,10]. The exact role of delta-toxin in the pathogenesis of necrotic enteritis has not been elucidated. Pore-forming toxins impair the barrier function of the intestinal epithelium [14,15,16]. Alpha-toxin from *S. aureus* disrupts the epithelial barrier function in human intestinal epithelial Caco-2 cells [15]. Alpha-toxin elevates a disintegrin and metalloprotease (ADAM) 10 activity in epithelial cells, resulting in the cleavage of E-cadherin, the key membrane protein of adherens junctions [17,18,19]. ADAM10 acts as a cellular receptor for alpha-toxin. We also previously reported that delta-toxin disturbed the barrier integrity of human intestinal epithelial Caco-2 cells [20]. Delta-toxin caused the activation of ADAM10, and ADAM10-mediated E-cadherin cleavage affected the intestinal epithelial barrier, suggesting that ADAM10 is involved in the intestinal impairment caused by the toxin [20]. However, the intestinal tissue damage induced by delta-toxin remains unknown.

The purpose of the present study was to examine the effects of delta-toxin on the mouse intestinal mucosa using an ileal loop model. In particular, we investigated the involvement of E-cadherin and ADAM10 in the toxin-induced pathological changes.

## 2. Results

### 2.1. Effect of Delta-Toxin on Fluid Accumulation in Mouse Intestinal Loops

**Figure 1:**
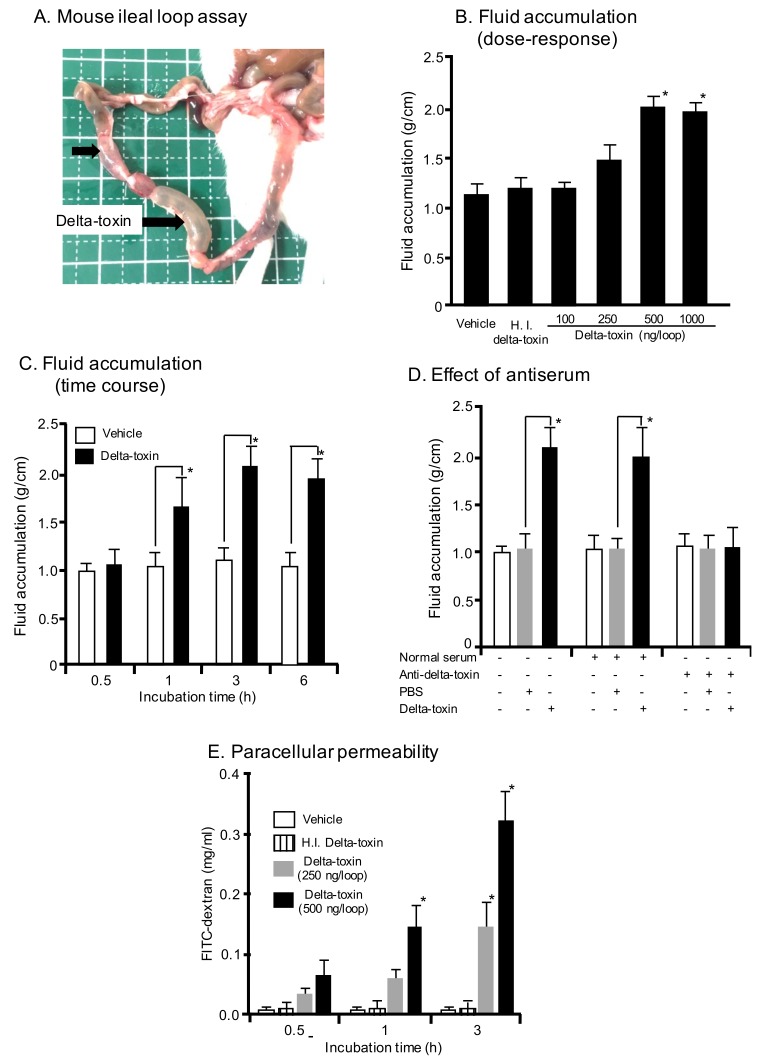
Mouse ileal loop test for delta-toxin. (**A**) Gross lesions of inoculated loops. A total of 0.1 mL of PBS containing trypsin inhibitor (TI) (vehicle) or PBS containing delta-toxin (250 ng) and TI were inoculated into mouse ligated ileal loops and then incubated for 3 h. (**B**) Fluid accumulation after 3 h treatment with various doses of delta-toxin or 5000 ng of heat-inactivated (HI) delta-toxin plus TI. (**C**) Fluid accumulation at various time points after inoculation with delta-toxin (500 ng/loop) plus TI. (**D**) Effect of anti-delta-toxin antibodies on delta-toxin-induced fluid accumulation. Ligated ileal loops were challenged for the indicated time periods with delta-toxin, delta-toxin plus rabbit anti-delta-toxin serum, delta-toxin plus normal rabbit serum, or PBS in the presence of TI. (**E**) Paracellular permeability. In vivo intestinal paracellular permeability was evaluated by measuring FITC-dextran in the blood after luminal inoculation of 4.4-kDa FITC-dextran and delta-toxin at various time points. After incubation, the blood was sampled by cardiac puncture to determine the plasma FITC-dextran concentration. Results indicate means ± SD (n = 6). * *p* < 0.01. One-way analysis of variance was employed to assess significance.

In the present study, we intend to study the effects of delta-toxin in a mouse ileal loop model. For this purpose, mouse ligated ileal loops were treated with delta-toxin or PBS control in the presence of trypsin inhibitor (TI). After delta-toxin treatment for 3 h, the loop injected with the toxin was swollen, and fluid accumulation was observed (Figure 1A). In contrast, the loop injected with PBS as a negative control (vehicle) did not exhibit any pathological changes. As shown in Figure 1B, the toxin (250–1000 ng/loop) dose-dependently caused fluid accumulation in mouse ligated ileal loops. Less than 100 ng/loop of delta-toxin induced no fluid accumulation. However, heat-inactivated delta-toxin exhibited no fluid accumulation at any dose. The time-course for fluid accumulation induced by the toxin was examined (Figure 1C). Fluid accumulation was observed within 1 h and reached a maximum within 3 h. To confirm that delta-toxin actively caused intestinal damage, we examined the effect of the preincubation of delta-toxin with an anti-delta-toxin antiserum. The fluid accumulation caused by the delta-toxin was neutralized by the antiserum against the delta-toxin but not by normal rabbit serum (Figure 1D). On the other hand, fluid accumulations in loops inoculated with delta-toxin without TI were similar to those discovered in PBS-inoculated loops (data not shown). It has been reported that delta-toxin disrupts the barrier function of Caco-2 cells, as evidenced by reduced transepithelial electrical resistance (TEER) and decreased cellular levels of adherence junction protein E-cadherin [20]. Concomitantly, the toxin induced a parallel increase in paracellular permeability. In order to confirm this, in vivo fluorescein isothiocyanate (FITC)-dextran trans-intestinal mobilization studies were carried out. FITC-dextran and delta-toxin were injected into the intestinal loops, and the serum levels of FITC-dextran were determined. As shown in Figure 1E, the delta-toxin dose- and time-dependently caused an increase in serum FITC-dextran level, as compared with the PBS controls. Taken together, these results indicated that the paracellular permeability of the mouse ileum was altered by delta-toxin exposure.

### 2.2. Histopathological Damage Caused by Delta-Toxin in Mouse Intestinal Loops

**Figure 2:**
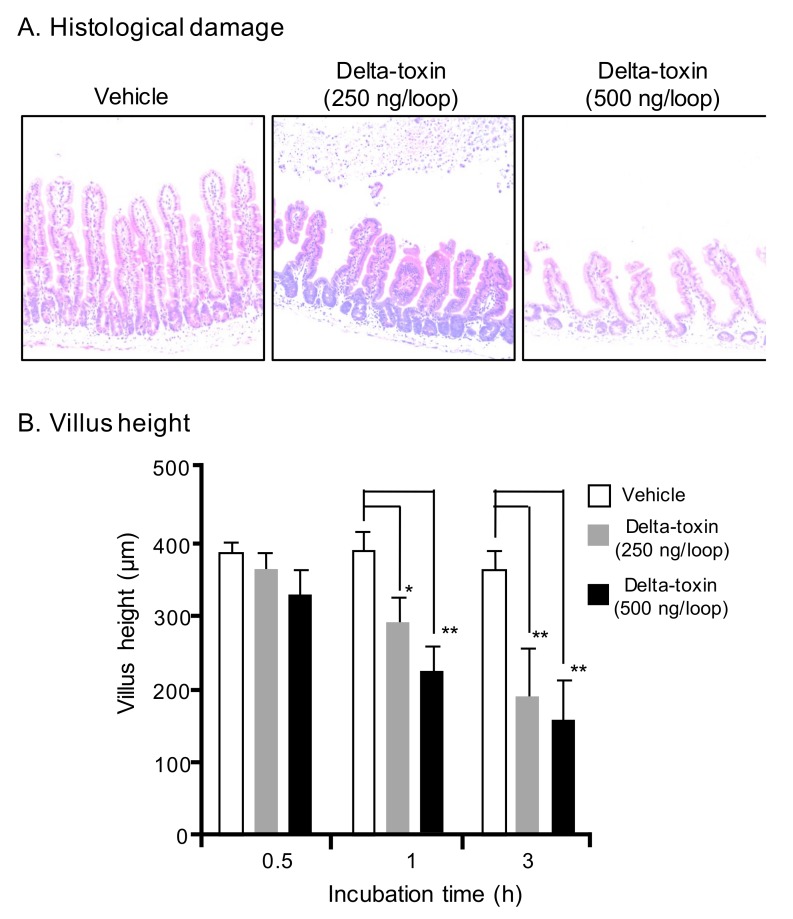
Delta-toxin-caused histological damage in the mouse small intestine. (**A**) Mouse ligated ileal loops were treated with PBS containing TI (vehicle), or PBS containing delta-toxin and TI for 3 h. The sections were stained with hematoxylin and eosin and photographed at a magnification of ×200. A typical photomicrograph from one of six experiments is shown. (**B**) Villus heights for ileum. The means ± standard deviation (SD) from ten experiments is indicated. * *p* < 0.05, ** *p* < 0.01. One-way analysis of variance was employed to assess significance.

To investigate whether delta-toxin causes intestinal tissue damage, the histological damage in the ligated ileal loops was examined. After a 3 h challenge, intestinal loops treated with 250 or 500 ng/loop of delta-toxin exhibited pathological changes compared with vehicle treated loops (Figure 2A). The overall severity of delta-toxin damage increased with the treatment dose. Since the shortening of the intestinal villus is generally employed as a measure of intestinal damage, we determined the villus heights after delta-toxin treatment. As shown in Figure 2B, delta-toxin caused a dose- and time-dependent shortening of villi in the loop. In the loop at 3 h, the mean villus height was reduced by about 50% compared with villi from vehicle-treated mice. In contrast, no intestinal damage was detected in any loops treated with delta-toxin in the absence of TI (data not shown). These results indicated that delta-toxin caused intestinal damage characterized by villus shortening and fluid accumulation.

### 2.3. E-Cadherin Degradation Induced by Delta-Toxin in Mouse Intestinal Loops

**Figure 3:**
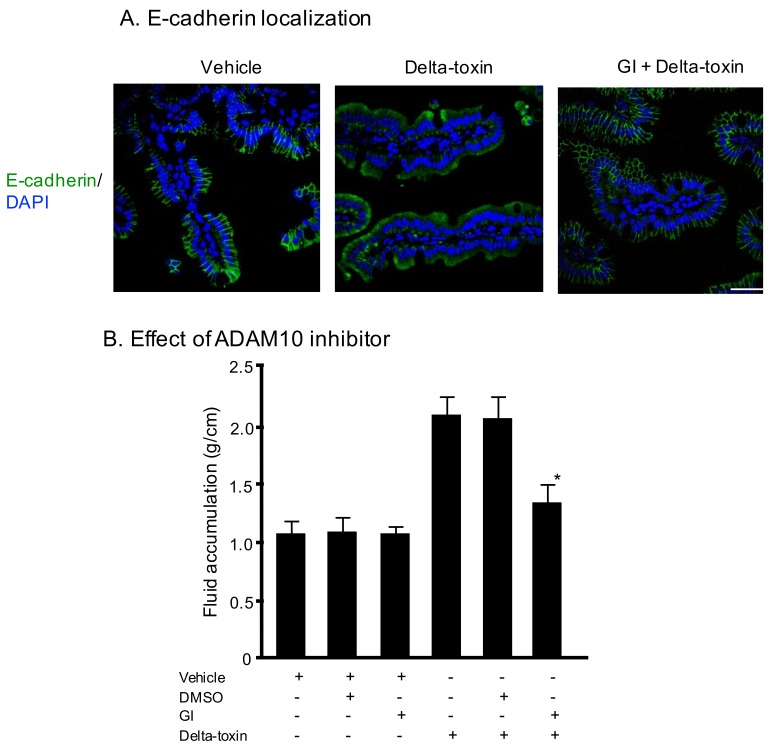
E-cadherin localization on sections of delta-toxin-treated ileum. (**A**) E-cadherin localization on sections of intestine from delta-toxin-treated mouse and control. E-cadherin staining (green) and 4′,6-diamidino-2-phenylindole (DAPI) staining (blue) were visualized with confocal immunofluorescence microscopy. Experiments were repeated three times, and representative data are shown. Scale Bars: 50 μm. (**B**) Mouse ligated ileal loops were treated with PBS containing 20 μM GI254023X or DMSO and TI, or PBS containing delta-toxin, 20 μM GI254023X or DMSO and TI for 3 h. The mean ± standard deviation (SD) from six experiments is indicated. * *p* < 0.01. One-way analysis of variance was employed to assess significance.

We previously reported that delta-toxin promotes ADAM10-mediated E-cadherin cleavage on Caco-2 cells [20]. Therefore, we examined E-cadherin localization using immunofluorescence staining of ileal tissue sections. As shown in Figure 3A (vehicle), E-cadherin was initially present at all cell–cell contacts. The E-cadherin staining of delta-toxin-treated tissue was almost undetectable. In contrast, in the presence of GI254023X, an inhibitor of ADAM10, the toxin-treated tissues showed E-cadherin at the cell–cell contacts. To investigate the role of E-cadherin in delta-toxin-induced fluid accumulation, we examined the effects of GI254023X. As shown in Figure 3B, the inhibitor inhibited the fluid accumulation caused by delta-toxin. These results indicate that the toxin caused E-cadherin degradation in mouse ileal tissues.

### 2.4. Delta-Toxin-Induced Cell Shedding from Small Intestinal Villi

**Figure 4:**
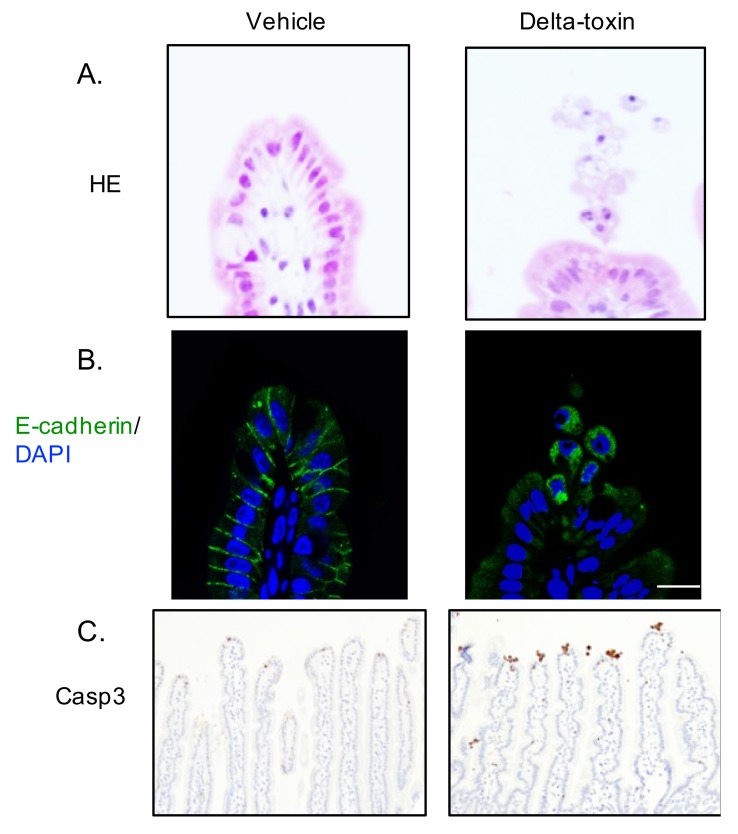
Delta-toxin-induced shedding of intestinal epithelial cells. Mouse ligated ileal loops were treated with PBS containing TI (vehicle), or PBS containing delta-toxin and TI for 1 h. (**A**) Hematoxylin and eosin stained sections were photographed. (**B**) E-cadherin staining (green) and DAPI stained sections were viewed using confocal immunofluorescence microscopy. (**C**) Immunostaining of intestinal epithelial cells for active caspase-3. Experiments were repeated three times, and representative data are shown. Scale Bars: 20 μm.

Intestinal epithelial cell shedding might be an initial indicator of epithelial cell damage in many intestinal injuries [21]. In the ligated ileal loops 1 h after delta-toxin administration, cell shedding from the villi was observed (Figure 4). This observation shows that delta-toxin rapidly induces small intestine villus epithelial lesions. Loss of E-cadherin in intestinal epithelial cells leads to cell shedding [22]. To assess the role of E-cadherin in delta-toxin-induced cell shedding, we carried out E-cadherin staining of delta-toxin-treated tissues. As shown in Figure 4, delta-toxin caused the loss of E-cadherin in intestinal epithelial cells. E-cadherin localized in the intracellular vesicles of shedding cells, indicating that the toxin induces the internalization of E-cadherin into shedding enterocytes. Next, caspase-3 is activated prior to pathologic cell shedding [23]. To study the involvement of caspase-3 in the toxin-induced cell shedding, we performed immunohistochemistry for active caspase-3 (Figure 4). After the administration of delta-toxin, active caspase-3 emerged at the tips of intestinal villi as early as 1 h, indicating that delta-toxin causes intestinal epithelial cell apoptosis.

## 3. Discussion

Delta-toxin produced by *C. perfringens* is well-known to possess cytolytic activity through promoting the formation of oligomeric transmembrane pores in the host cell surface [1,10,11]. Delta-toxin can alter the permeability of cytoplasmic membranes and phospholipid bilayer membranes via membrane insertion [6,7,9,12]. Recently, we reported that ADAM10 plays an important role in toxin-induced cytotoxicity [20]. In the current study, we showed that E-cadherin degradation by delta-toxin-activated ADAM10 is involved in intestinal epithelial cell damage.

*C. perfringens* type B and type C strains cause necrotizing enteritis in domestic animals and humans [1,2,3,4]. These strains produce beta-toxin and delta-toxin. It has been reported that beta-toxin is the main pathogenic factor of type B and C strains [4,13]. However, the role of delta-toxin in infectious disease has remained poorly characterized. The current study indicated, for the first time, that purified delta-toxin alone elicits dose- and time-dependent fluid accumulation and histological damage in mouse ileal loops. The fluid accumulation caused by delta-toxin in this study could be neutralized by an anti-delta-toxin antibody, confirming delta-toxin’s involvement in the observed fluid accumulation. We demonstrated that delta-toxin has enterotoxic activity. Measurement of the serum appearance of FITC-dextran (4.4 kDa) was classically utilized to determine the migration of small molecules across the intestinal epithelia in vivo. Here, we showed that delta-toxin increases intestinal permeability to FITC-dextran in a time- and dose-dependent manner, indicating that the toxin elevates intestinal epithelial paracellular permeability. The time-course manner of delta-toxin-induced fluid accumulation was similar to that of the intestinal permeability of FITC-dextran by the toxin. Moreover, the present results indicate that the toxin-induced elevation of intestinal permeability is accompanied by a histological change in the intestinal epithelia. Therefore, the intestinal damage is induced by the direct effects of the delta-toxin on the intestinal cells. We have previously reported that delta-toxin disrupts the intestinal epithelial barrier function in Caco-2 cell monolayers [20]. Collectively, our findings indicate that the intact intestinal epithelial barrier function is disrupted after treatment with delta-toxin.

Strains of *C. perfringens* type C are involved in necrotic enteritis in humans [1,2,3,4]. The important factors leading to development of the disease include low protein diets, which are a leading cause of low trypsin generation; pancreatic disorders; and intakes of diets containing trypsin inhibitors [1,4,13]. These factors play a role in the long-lasting action of beta-toxin in the small intestine, which prevents the toxin degradation by endogenous trypsin [1,4,13]. In this study, we found that delta-toxin caused the intestinal damage in the presence of TI but not in the absence of TI. The necessity of TI in delta-toxin-induced enteric injury reflects natural type C infection in humans and animals. The damaging effect of beta-toxin on the ileal loop was observed at 1–10 μg [3,13]. In the present study, delta-toxin caused ileal injury at a dose of 250–500 ng. Delta-toxin caused more damaging effects than beta-toxin. Thus, delta-toxin plays a role in type C infection.

In the current study, we observed that delta-toxin causes villus shortening coincident with the histological damage. Villus shortening is associated with the shedding of intestinal epithelial cells [24]. After the treatment of ligated ileal loops with delta-toxin, intestinal epithelial cells at the villus tips detached and separated from adjacent cells with a teardrop-like form. These results indicated that the delta-toxin-induced cell shedding occurred simultaneously with villus shortening. Moreover, we showed that the shedding cells contained activated caspase-3, a marker of apoptosis. In various enteric diseases, increased villus epithelial cell apoptosis leads to pathological intestinal epithelial cell shedding [21]. It has been reported that apoptotic cells in the villus deform and slowly separate from the surrounding cells for apoptotic epithelial clearance [25]. Therefore, we concluded that delta-toxin induced intestinal epithelial cell apoptosis and shedding, and that this triggered villus shortening. These observations showed that delta-toxin caused cell shedding, which correlated with fluid exudation into the intestinal lumen.

We previously reported that delta-toxin provokes intestinal epithelial barrier dysfunction through the cleavage of E-cadherin following ADAM10 activation [20]. E-cadherin is a principal constituent of adherens junctions. Loss of E-cadherin in the small intestine has been correlated with intestinal epithelial barrier dysfunction and homeostasis, leading to apoptosis and cell shedding [22]. We found that the toxin caused a loss of E-cadherin in the intestinal epithelial cells, which was inhibited by ADAM10 inhibitor, and evoked E-cadherin internalization in the cytoplasmic vesicles of shedding cells. Loss of anchorage causes the internalization of E-cadherin through an endocytic pathway [23]. Therefore, delta-toxin-induced ADAM10 activation induces E-cadherin internalization, resulting in increased accumulation of E-cadherin in cytoplasmic vesicles. Delta-toxin belongs to the same group as the *S. aureus* alpha-toxin. It has been reported that alpha-toxin activates ADAM10 activity in alveolar epithelium, leading to cleavage of the E-cadherin [18]. Moreover, this cleavage plays a role in the toxin-induced destruction of the epithelium, serving as the virulence of acute lung injury [18]. Thus, we think that delta-toxin acts by the same mechanism as the alpha-toxin. Taken together, our data demonstrate that a delta-toxin-induced loss of E-cadherin triggers the intestinal pathological process.

## 4. Conclusions

The present results showed that delta-toxin induced intestinal epithelium injury caused by the loss of E-cadherin digested by ADAM10 activation. This information will improve the understanding of the molecular mechanisms of delta-toxin-induced intestinal pathogenesis.

## 5. Materials and Methods 

### 5.1. Materials

Recombinant delta-toxin and rabbit anti-delta-toxin antibody were prepared as described previously [13]. Fluorescein isothiocyanate (FITC)–dextran (average mol wt 3000–5000), GI254023X, 10% neutral buffered formalin, and hydrogen peroxide were purchased from Merck (Tokyo, Japan). Rabbit anti-N-terminal fragment of E-cadherin antibody and normal rabbit IgG as an isotype control were obtained from Santa Cruz Biotechnology (Santa Cruz, CA, USA). Trypsin inhibitor (TI), 3,3′-diaminobenzidine, HistoVT One, and Hanks’ balanced salt solution (HBSS) were obtained from Nacalai Tesque (Kyoto, Japan). Rabbit anti-cleaved caspase-3 was purchased from Cell Signaling Technology (Tokyo, Japan). A peroxidase-labeled anti-rabbit EnVision™ secondary antibody was obtained from Dako (Cambridge, UK). Alexa Fluor 488-conjugated goat anti-rabbit IgG and 4′,6-diamidino-2-phenylindole (DAPI) were obtained from ThermoFisher (Tokyo, Japan). All other chemicals were of the highest grade available from commercial sources.

### 5.2. Mice

Experimental studies were carried out utilizing male, 25–30 g, Slc:ICR mice after a 1-week acclimatization period. The mice were obtained from Japan SLC, Inc. (Shizuoka, Japan) and were kept in a light and temperature-controlled facility with free chow and water intake at Tokushima Bunri University. Animal experiments were approved by the Animal Care and Use Committee of Tokushima Bunri University (code: #17-4; date of approval: 20 April 2017), and all procedures were performed in accordance with institutional guidelines, which conform to the Fundamental Guidelines for Proper Conduct of Animal Experiment and Related Activities in Academic Research Institutions under the jurisdiction of the Ministry of Education, Culture, Sports, Science and Technology, 2006.

### 5.3. Mouse Loop Assay

Male, 25–30 g, SLC:ICR mice were used. Mice were fasted overnight with free access to water before inoculation. Mice were placed in an induction chamber with inhalation anesthesia apparatus (Narcobit-E type II, Natsume Seisakusho, Tokyo, Japan) for anesthetic induction with 3% isoflurane (Wako Pure Chem. Ind. Ltd., Osaka, Japan). Since C. perfringens beta-toxin can induce intestinal lesions in ligated intestinal loops in the presence of trypsin-inhibitor (TI) [13], we performed the ligated ileal loop tests of delta-toxin in the presence of TI. Anesthesia was maintained with 3% isoflurane delivered using a facemask and inhalation anesthesia apparatus. A midline laparotomy was carried out to exteriorize the small intestine. The ileum was ligated with surgical silk threads approximately 3–4 cm in length, each with empty interloops to prevent any cross-contamination between loops. The loops were inoculated with 0.1 mL of PBS plus trypsin inhibitor (TI, 150 μg/mL) or delta-toxin plus TI (150 μg/mL). In some studies, delta-toxin plus TI were incubated for 60 min at 37°C with rabbit anti-delta-toxin serum or rabbit normal serum. Similarly, heat-inactivated (HI) delta-toxin samples were prepared by boiling for 10 min, and then HI delta-toxin was mixed with TI and inoculated. The ligated loops were then returned to the abdominal cavity, the incision was closed by separate muscle and skin sutures, and the animals were allowed to regain consciousness. After the assigned treatment periods, mice were sacrificed, the ligated loops were removed, and the weights and lengths of the loops were measured. The isolated loops were then cut open to eliminate luminal fluid before the loops were re-weighed. The difference in weight before and after luminal fluid elimination was utilized to determine the loop weight-to-length ratio (g/cm) of fluid accumulation.

### 5.4. Paracellular Permeability

Paracellular intestinal permeability was assessed utilizing the measurement of a macromolecular marker: 0.1 mL of PBS (pH 7.2) containing 25 mg of FITC-dextran and TI (150 μg/mL) or 25 mg of FITC-dextran and TI (150 μg/mL). Beta-toxin was inoculated into the ligated ileal loops, and then the abdominal laparotomy was sutured [26]. At designated time points, blood was collected by cardiac puncture. The blood was centrifuged at 3500 *g* for 15 min (4°C), and the serum was collected for determination of the concentration of FITC-dextran utilizing a fluorospectrophotometer (Tecan Infinite^®^ 200 PRO, Kawasaki, Japan) with 480 nm excitation and 520 nm emission filters. The amount of FITC-dextran in plasma was calculated from standard curves generated by fluorometric measurements of FITC-dextran at known concentrations.

### 5.5. Histological Analysis

For histological analysis, intestinal samples were fixed in 10% neutral buffered formalin, dehydrated through graded alcohol solutions to xylene, and embedded in paraffin. Tissue sections were cut at 5 μm and stained conventionally with hematoxylin and eosin. Sections were then photomicrographed using a Nikon microscope (Tokyo, Japan) at ×200 magnification. Measurements of the villus height (from the top of the villi to the villus-crypt junction) were performed under a light microscope at ×100 magnification (Nikon microscope). Ten intact, well-oriented villi and crypts were measured and averaged for each sample.

### 5.6. Immunohistochemistry

For the fluorescent immunohistochemical study, the ileal tissues were removed, washed in ice-cold PBS, fixed in 4% paraformaldehyde, embedded in Tissue-Tek OCT (Sakura FineTek Japan, Tokyo, Japan), and frozen rapidly in liquid nitrogen. Cryosections (Leica CM1850, Wetzlar, Germany) were cut serially into 7-μm sections and mounted on silane-coated slides (Matsunami, Osaka, Japan). The slides were incubated for 24 h at room temperature with a rabbit anti-N-terminal fragment of E-cadherin antibody and for 2 h at room temperature with an Alexa Fluor 488-labeled donkey polyclonal anti-rabbit IgG antibody, respectively. DAPI was utilized to visualize nuclei. Immunofluorescence was observed using a Nikon A1 confocal laser scanning microscope (Tokyo, Japan). Images indicated in the figures are representative of at least four independent experiments.

### 5.7. Statistical Analysis

The statistical test was performed with Easy R (Saitama Medical center, Jichi Medical University, Saitama City, Japan) [27]. Using multiple comparison methods, one-way analysis of variance (ANOVA) was used followed by Tukey’s test. Differences between the two groups were analyzed using the two-tailed Student t-test. *p*-values of 0.05 or less were considered significant.
